# Sphingomyelinases D From *Loxosceles* Spider Venoms and Cell Membranes: Action on Lipid Rafts and Activation of Endogenous Metalloproteinases

**DOI:** 10.3389/fphar.2020.00636

**Published:** 2020-05-13

**Authors:** Priscila Hess Lopes, Carmen W. van den Berg, Denise V. Tambourgi

**Affiliations:** ^1^Laboratório de Imunoquímica, Instituto Butantan, São Paulo, Brazil; ^2^Centre for Medical Education, School of Medicine, Cardiff University, Cardiff, United Kingdom

**Keywords:** lipid rafts, *Loxosceles*, Sphingomyelinases D, ADAMs, proprotein convertases

## Abstract

*Loxosceles* spider venom contains Sphingomyelinase D (SMase D), the key toxin causing pathology. SMase D hydrolyzes the main component of lipid rafts, sphingomyelin, which changes the membrane microenvironment resulting in the activation of endogenous metalloproteinase from the ADAMs family. Alterations in membrane microenvironment of lipid rafts contribute to the activation of several cell surface molecules. Serine proteinases convertases acting on the pro-domain of membrane metalloproteinases, such as ADAMs, increase the cleavage and the release of proteins ectodomains and receptors located at the cell surface areas containing lipid rafts. We, therefore, investigated the interaction of SMases D with these membrane microdomains (lipid rafts) in human keratinocytes, to better understand the molecular mechanism of SMases D action, and identify the ADAM(s) responsible for the cleavage of cell surface molecules. Using specific inhibitors, we observed that ADAMs 10 and 17 are activated in the cell membrane after SMase D action. Furthermore, proproteins convertases, such as furin, are involved in the SMase D induced ADAMs activation. One of the signaling pathways that may be involved in the activation of these proteases is the MAPK pathway, since phosphorylation of ERK1/2 was observed in cells treated with SMase D. Confocal analysis showed a strong colocalization between SMase D and GM_1_ ganglioside present in rafts. Analysis of structural components of rafts, such as caveolin-1 and flotillin-1, showed that the action of SMase D on cell membranes leads to a reduction in caveolin-1, which is possibly degraded by toxin-induced superoxide production in cells. The action of the toxin also results in flotilin-1 increased detection in the cell membrane. These results indicate that SMases D from *Loxosceles* venoms alter membrane rafts structure, leading to the activation of membrane bound proteases, which may explain why the lipase action of this toxin can result in proteolytic cleavage of cell surface proteins, ultimately leading to pathology.

## Introduction

*Loxosceles* spiders envenomation (Sicariidae Family) occur in temperate and tropical regions of North, Central, and South America, Africa, Asia, and Europe (Wasserman and Anderson, 1983; Platnick, 2011). Bites by these spiders commonly result in local necrotic skin lesions and more rarely cause systemic effects including hemolysis, intravascular coagulation, and thrombocytopenia, which may result in renal failure (Barretto et al., 1985; Schenone et al., 1989; Tambourgi et al., 1998).

Forrester et al. (1978), analyzing *Loxosceles reclusa* venom, showed the association of venom toxicity with sphingomyelinase activity, and sphingomyelinase D (SMase D) is now considered the most important component for the establishment of this spider envenomation pathology (Tambourgi et al., 1998). We previously showed that SMases D from *Loxosceles* venom induced activation of membrane-bound metalloproteinases from the Adamalysin family, by indirect action on the cell surface in a variety of cells (Tambourgi et al., 2000; van den Berg et al., 2002). This resulted in e.g. the cleavage and ectodomain shedding of Glycophorins (GPs), endothelial protein C receptor (EPCR), and Thrombomodulin (TM), explaining the observed complement mediated hemolysis and intravascular coagulation (Tambourgi et al., 2000; van den Berg et al., 2002; Paixão-Cavalcante et al., 2006). In addition, we demonstrated that SMase D induces the ADAM (ADAM: a desintegrin and metalloprotease) mediated ectodomain shedding of numerous other cell surface molecules including MCP (Membrane Cofactor Protein: MCP; CD46), Major Histocompatibility Complex class I (MHCI), β2-microglobulin (associated with MHCI), Epidermal Growth Factor Receptor (EGFR), and the C5a receptor (CD88) in many cell types, including keratinocytes (reviewed by [Tambourgi et al., 2010]). We have used keratinocytes successfully as a model to study the molecular mechanisms operating in cutaneous loxoscelism (Paixão-Cavalcante et al., 2006; Paixão-Cavalcante et al., 2007; Corrêa et al., 2016; Lopes et al., 2019).

ADAMs are transmembrane proteases belonging to the family of Metzicins, subfamily of Adamlysins. They induce ectodomain shedding of a number of cell surface proteins and are considered crucial in modulating various physiological and pathophysiological processes (van Goor et al., 2009). The mechanism by which the *Loxosceles* venom induces activation of these ADAMs is not yet understood.

The metalloprotease domain of ADAMs is protected by a pro-domain and the primary pathway of activation and removal of the pro-domain is performed by proprotein convertases (PCs) such as furin, PC7, PC5/6B, and SKI-1 (Seidah, 2006; Klein and Bischoff, 2011). These proprotein convertases belong to a family of serine proteinases of the Subtilisins type (Seidah et al., 2008) and play an important role in the regulation of ADAMs (Reviewed by [Seals and Courtneidge, 2003]). Several studies showed that inhibition of furin transport from the Golgi to the cell membrane, by Brefeldin A and monensin, resulted in a decrease in activity of ADAM-17 (Lum et al., 1998; Roghani et al., 1999; Howard et al., 2000; Kang et al., 2002). Overexpression of PC7 increased the activity of ADAM-10 (Anders et al., 2001), and the genetic modification of the furin binding site of ADAMs 10, 12, and 19 prevented their activation (Loechel et al., 1998; Anders et al., 2001; Kang et al., 2002).

The shedding of ectodomains of surface molecules by ADAMs proteins may occur or increase due to various cellular stimuli (Walev et al., 1996; Müllberg et al., 2000; Chalaris et al., 2007), including those that result in the activation of MAPK and ERK signaling pathways (Xu et al., 2012). Furthermore, the cleavage and release of ectodomains are influenced by the spatial organization of the transmembrane molecule and protease within the lipid microenvironment of membranes (Walev et al., 2000; Kojro et al., 2001; Matthews et al., 2003; von Tresckow et al., 2004; Zimina et al., 2007). Maturation of ADAM-17 occurs in lipid rafts and the mechanisms that regulate the hydrolytic activity of this protease, on various substrates, involve the re-distribution of the target proteins within the lipid rafts (Walev et al., 2000; Kojro et al., 2001; Matthews et al., 2003; von Tresckow et al., 2004; Zimina et al., 2007).

Lipid analysis has revealed that over 70% of all cellular sphingomyelin (SM), the main substrate for *Loxosceles* SMase D, is located in lipid rafts (Smart et al., 1999) and that SM, as well as other sphingolipids, play an important role in the physical properties of biological membranes (Giocondi et al., 2004), and are necessary to maintain the integrity of the lipid rafts. A sphingomyelinase, from *Staphylococcus aureus*, altered the properties of lipid rafts in peripheral blood derived mononuclear cells, resulting in a concomitant reduction of cholesterol content of the rafts (Diaz et al., 2005). In addition, the composition and function of membrane rafts can be modulated in response to a number of factors and conditions (Simons and Ikonen, 1997) including Reactive Oxygen Species production (Park et al., 2009; Mougeolle et al., 2015) and ceramide generation (Zhang et al., 2009) and their functions are closely related to the associated proteins.

Considering that (*i*) interference with organization of lipid rafts or SM-hydrolysis can lead to changes in various biological processes in the cell, (*ii*) that shedding of cell surface molecules depends on the membrane microenvironment and (*iii*) that SMases D in the venoms of *Loxosceles* hydrolyze SM, we aimed to investigate the effects of *Loxosceles* SMase D on the activation of metalloproteinases, proprotein convertases, and lipid raft structure in human keratinocytes, in order to elucidate the complex action of this toxin.

## Material and Methods

### Reagents, Antibodies, and Buffers

Broad spectrum matrix metalloprotease inhibitor Galardin (GM6001) (Li et al., 2002), proprotein convertases inhibitors, FI (Furin inhibitor); FII (Furin, PACE4 and PC1 inhibitor); ProproC (Furin, PACE4, PC1/3, PC4, and PC5/6 inhibitor) were obtained from Merck-Millipore (Darmstadt, Germany). Specific inhibitors for ADAM-10 (GI254023 abbreviated GI) and ADAM17 (GW280264; abbreviated GW) (Ludwig et al., 2005) were kindly provided by Prof. Ann Ager (Cardiff University, UK). PMSF (serineprotease inhibitor) and Monensin (Golgi transport inhibitor), Bovine serum albumin (BSA), paraformaldehyde were purchased from Sigma Aldrich (St. Louis, MO, USA). Dimethyl sulfoxide (DMSO) and Tween-20 were obtained from Merck-Millipore (Darmstadt, Germany), “Prolong Gold antifade” containing 4',6-diamidino-2-phenylindole (DAPI) nuclear stain was from Invitrogen (Paisley, UK). EIA Titerzime Phospho-ERK1/2 Enzyme Immunometric Assay was from Assay Designs (Ann Arbor, MI, USA). Reagents for analysis of ROS and RNOS production, Dihydroethidium (DHE) and Dihydrorhodamine-123 (DHR) respectively, were obtained from Sigma-Aldrich (MO, USA) and Alexa555-conjugated Cholera Toxin subunit b (CTx-b/Alexa555)-was obtained from Molecular Probes (Eugene, Oregon, USA). Rabbit antibodies against Flotilin-1 and Caveolin-1, FITC-conjugated secondary antibodies as rabbit anti-mouse IgG (RAM/FITC) or goat anti-rabbit IgG (GAR/FITC) were obtained from Sigma-Aldrich (Saint Louis, MI, USA). Mouse monoclonal antibodies (MoAbs) against human EGFR (Epidermal Growth Factor Receptor), MCP (CD46: Membrane Cofactor Protein), β_2_-microglobulin, TNF-RI (CD120a: Tumor Necrosis Factor–Receptor 1), and streptavidin-PE were purchased from BD Biosciences (San Jose, CA, USA). MoAbs against human ADAM-17 and ADAM-10, rabbit IgG against GM_1_ ganglioside, and Goat anti-rabbit conjugated Alexa 488 (GAR/Al488) were from Abcam (Cambridge, UK). MoAb against human CD59 (Bric229) was from International Blood Group Reference Laboratory (IBGRL, Bristol, UK). Rabbit IgG anti-*Loxosceles* SMase D was produced in house. DMEM (Dulbecco's Modified Eagle Medium) and penicillin-streptomycin were purchased from Gibco, Invitrogen Corp. (Eugene, Oregon, USA), and Fetal bovine serum (FBS) was from Cultilab (São Paulo, Brazil). ATV (Trypsin 0.2% and Versene 0.02%) was purchased from Adolpho Lutz Institute (São Paulo, Brazil). Buffers: Veronal buffered saline–VBS^2+^ (2.8 mM barbituric acid, 145.5 mM NaCl, 0.8 mM MgCl_2_, 0.3 mM CaCl_2_, 0.9 mM Na-barbital, pH 7.2), Phosphate buffered saline–PBS (8.1 mM Na_2_HPO_4_; 1.5 mM KH_2_PO_4_; 137 mM NaCl; 2.7 mM KCl, pH 7.4), FACS buffer (PBS buffer containing 1% of albumin and 0.1% of sodium azide), and FACS fixing solution (FACS buffer containing paraformaldehyde 1%).

### Expression and Purification of Recombinant Sphingomyelinase D

The recombinant Sphingomyelinase D (SMase D) from *L. laeta* venom was prepared as described by Fernandes-Pedrosa et al. (Fernandes Pedrosa et al., 2002). The permission to access to genetic resources register n° AEE9AEA 11/01/2018 was provided by National System of Management of Genetic Heritage and Associated Traditional Knowledge (SisGen).

### Cells Culture

Human keratinocyte cell line HaCaT (obtained from Banco de Células do Rio de Janeiro - BCRJ) were grown in 75 cm^2^ flasks (Corning Inc., New York, USA) in DMEM supplemented with 10% fetal bovine serum and 1% penicillin-streptomycin, at 37°C and 5% CO_2_.

### Treatment of Cells

HaCaT cells were trypsinized with ATV and resuspended in VBS^2+^ buffer. Cells (1 × 10^6^ cells/ml), pre-incubated for 5 min with Galardin (GM6001, 90 µM), ADAM-10 (GI, 45 µM) or ADAM-17 (GW, 45 µM) inhibitors, PMSF (1 mM), Monensin (10 µg/ml); proprotein convertases inhibitor (FI, FII and ProproC, 20 µM) or their vehicles, were further incubated with SMase D (25 µg/ml) or buffer, for 2 h at 37°C under slight agitation.

### Cell Surface Markers Analyses

Cells treated as described in *Treatment of Cells*, were incubated for 30 min at 4°C with monoclonal antibodies against EGFR (1:200), MCP (1 µg/ml), β2-microglobulin (1:200), CD59 (1:250), ADAM-10 (1:1,000), ADAM-17 (1:100), GM_1_ ganglioside (1:50), Flotilin-1 (10 µg/ml), Caveolin-1 (8 µg/ml), and rabbit anti *Loxosceles* SMase D IgG (1:200). After washing, cells were incubated for 30 min at 4°C with secondary antibodies (RAM/FITC or GAR/FITC, 1:100). Some cells were also incubated for 30 min at 4°C with anti-TNF-RI biotin-labeled antibody (1:50), washed and, then, incubated with streptavidin-PE (1:200). Fluorescence intensity of 10,000 cells was analyzed in a flow cytometer (FACSCanto, Becton Dickinson, CA, USA).

### Analysis of the Production of Reactive Oxygen (ROS) and Reactive Nitrogen Oxide Species (RNOS) and Intracellular Signaling Pathway Activation in Human Keratinocytes

Analysis of RNOS and ROS production by human keratinocytes treated with SMase D was analyzed by flow cytometry. HaCaT (10^6^ cells) were treated with SMase D or buffer and incubated with 5 µmol/L of DHE (for superoxide, O2^•-^), or DHR (for peroxynitrite, ONOO^-^) for 1 h at 37 and 30°C, respectively and 5% CO_2_. Cells were spun (1,500 rpm, 5 min), and resuspended in 300 µl FACS fixing solution and fluorescence intensity was measured in the flow cytometer (FACScanto, Becton Dickinson, CA, USA).

Activation of pERK1/2 by SMase D was analysed using the EIA Titerzime Phospho-ERK1/2 Enzyme Immunometric Assay kit, according to the manufacturer's recommendations. The reaction was read in a plate reader (Multiskan-EX, Labsystems, Helsinki, Finland) at a wavelength of λ 450 nm. The calculation of the pERK1/2 concentration in the samples was performed using the pERK recombinant standard curve (62.5 to 2,000 pg/ml) with subtraction of the value of the blank. Protein concentration of cell lysates was determined by the method of Lowry et al. (1951).

### Analysis of SMase D Binding to Lipid Rafts

HaCaT cells (1.5 × 10^4^/well) were cultured in four well Culture slides (BD) for 24 h in complete medium, followed by culture for 24 h in serum free medium. Cells were treated with SMase D (5 µg/ml) or buffer in serum free medium for 2 h at 37°C and 5% CO_2_. Wells were washed five times using serum free medium, followed by incubation in PBS containing 5% BSA for 1 h at room temperature and brief washing with PBS for 5 min.

Binding of SMase D to the cells was assessed by incubation with rabbit IgG anti-SMase D (1:200 PBS/2% BSA, 30 min, RT), followed by three washes with PBS/Tween20 (0.01%) and incubation with GAR/Al488 (1:500 in PBS/2% BSA, 30 min, RT).

For the visualization of lipid rafts, cells were incubated with Alexa555-conjugated Cholera toxin subunit b (CTx-b/Al555) (1.3 µg/200 µl, 30 min, RT) (Harder et al., 1998; Gniadecki et al., 2002). Cells were washed three times in PBS/Tween20 (0.01%) and fixed in 1% paraformaldehyde for 20 min at 37°C. Slides were counterstained with “Prolong Gold antifade” containing DAPI nuclear stain and covered with coverslips for microscopy.

The effect of SMase D treatment on the colocalization of CD59 and ADAM-17 with GM_1_, in lipid rafts, was investigated by incubating the cells with MoAbs against human CD59 (1:250) or anti-human ADAM-17 (10 µg/ml). The colocalization of SMase D, CD59, and ADAM-17 proteins and CTx-b was assessed using BioImageXD v1.0 software (Kankaanpää et al., 2012) and threshold values were calculated by the method of Costes (Costes et al., 2004). Colocalization coefficients, according to Manders (Manders et al., 1993), were chosen since they represent the true degree of colocalization. M1 denotes the colocalization index of the green with red marking and M2 the colocalization index of the red with green marking. An average of ten different images, in three different focal planes, were analyzed *per* experiment in four independent experiments.

Alternatively, to evaluate whether the labeling was restricted to the membrane, cells were detached and treated with SMase D or buffer, in suspension for 2 h at 37°C followed by cytospin centrifugation (400 rpm for 5 min) (Cytospin 4, Thermo Scientific, Massachusetts, USA) to fix the cells to the microscopy slides and then incubated with antibodies as described above.

The photomicrographs were acquired in laser scanning confocal microscopy system (LSCM) (LSM 510 meta, Carl Zeiss, Jena, Germany) using objective C-Apochromat 63×/1.2W corr.

### Statistical Analysis

Data were expressed as mean ± standard error and statistically analyzed with GraphPad Prism, version 6.1 for Windows (San Diego, USA). Comparisons between more than two groups in relation to one variable were performed by One Way ANOVA and multiple comparisons performed by *post-hoc* Tukey HSD. Comparison of two or more variables between more than two groups was performed using Two Way ANOVA followed by *post-hoc* Bonferroni test. Comparisons between two groups were performed by Student's t test.

## Results

### ADAM-10 and 17 Are Activated as Consequence of SMase D Action on Keratinocyte Cell Membrane

**Figure 1:**
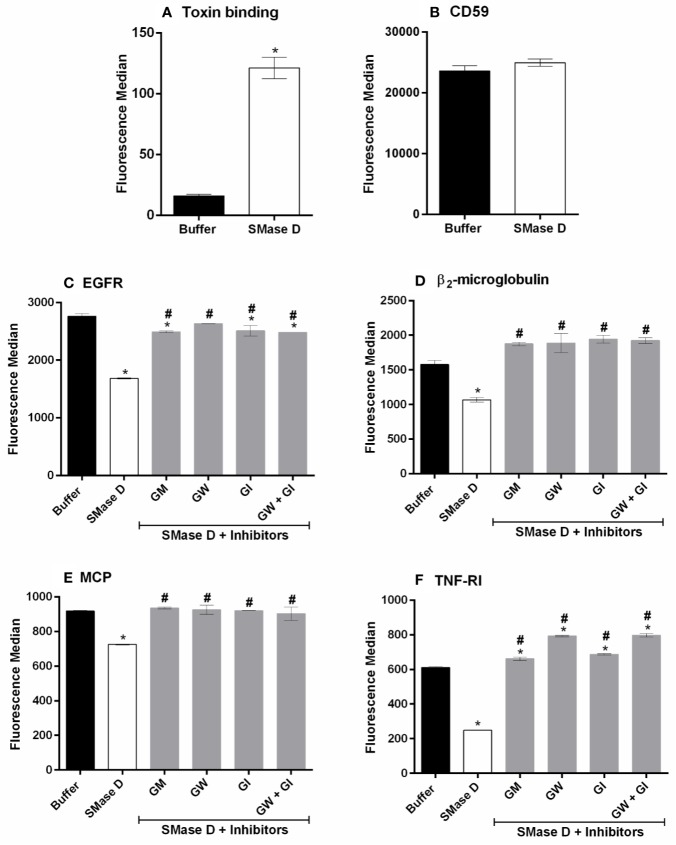
Involvement of ADAMs in the shedding of surface markers, induced by SMase D. HaCaT cells were pre-incubated for 5 min with Galardin (GM: 90 µM), ADAM-10 (GW: 45 µM), or ADAM-17 (GI: 45 µM) inhibitors followed by SMase D (25 µg/ml) or buffer for 2 h. Binding of the SMase D to keratinocytes surface **(A)**, Expression of CD59 in cells treated or not with SMase D **(B)**, EGFR **(C)**, β2-microglobulin **(D)**, MCP **(E)**, and TNF-RI **(F)** cell surface expression was analyzed by flow cytometry. Data are presented as mean ± standard error of duplicates being representative of two independent experiments. Statistically analyzed by one-way ANOVA, followed by Tukey's HSD test or t Test of Student in case of CD59 and toxin binding, using GraphPad Prism 6.1 Software. (*) Significant difference compared to buffer (p < 0.05). (#) significant difference in relation to SMase D (p < 0.05).

To investigate the role of ADAMs in the SMase D induced shedding of cell surface molecules from the human keratinocyte cell line HaCaT, the effects of a broad spectrum metalloproteinase inhibitor (Galardin: GM) and specific inhibitors of ADAM-10 (GI) and ADAM-17 (GW) were investigated. **Figures 1B–F** show that SMase D treatment of HaCaT cells resulted in reduced surface expression, likely as a consequence of shedding, of the membrane bound molecules EGFR, β2-microglobulin (associated with MHCI), MCP, and TNF-RI, but not CD59. This was significantly inhibited by the pre-incubation with GM and also by GI or GW, as well as a combination of both. This demonstrates that ADAMs-10 and -17 are activated as consequence of the action of SMase D from *Loxosceles* on cell membranes. As previously described by us (Paixão-Cavalcante et al., 2006), using anti-*Loxosceles* SMases D specific antibodies we could observe that the toxin binds to human keratinocytes membrane (**Figure 1A**).The use of ADAM inhibitors did not affect the cell viability nor the binding of the toxin to the cell membrane and the vehicle of the inhibitors did not produce any interference with the cleavage of the markers (data not shown).

### Proprotein Convertases Participate of the ADAMs Activation After SMase D Action on Keratinocyte Cell Membrane

**Figure 2:**
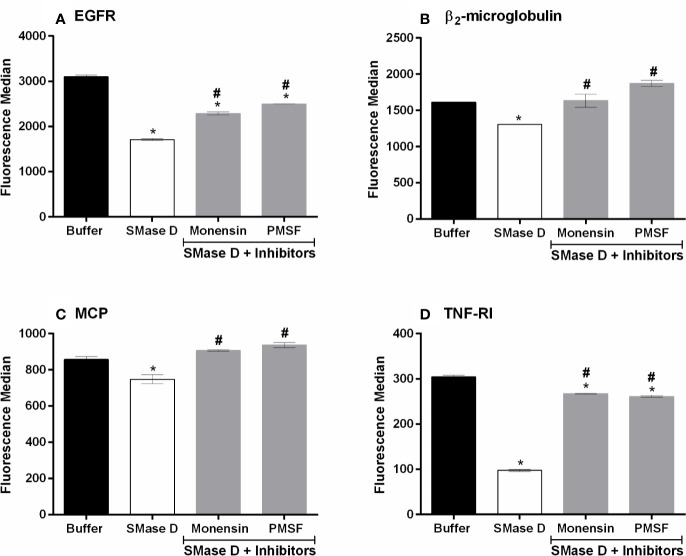
PMSF and Monensin inhibit the SMase D-induced shedding of surface markers. HaCaT cells were pre-incubated for 5 min with PMSF (1 mM) or Monensin (10 µg/ml) followed by SMase D (25 µg/ml) or buffer for 2 h. Expression of EGFR **(A)**, β2-microglobulin **(B)**, MCP **(C)**, and TNF-RI **(D)** was analyzed by flow cytometry. Data are presented as mean ± standard error of duplicates, representative of two independent experiments. Statistically analyzed by one-way ANOVA followed by Tukey HSD test using GraphPad Prism 6.1 Software. (*) Significant difference compared to buffer (p < 0.05). (#) Significant difference in relation to SMase D (p < 0.05).

ADAMs are zymogens and require cleavage of the prodomain, by certain serine proteinases, also known as proprotein convertases, such as furin (Seidah, 2006; Klein and Bischoff, 2011). In order to investigate whether furin is involved in the SMase D induced ADAMs activation, HaCaT cells were pre-treated with the broad-spectrum serine proteinase inhibitor PMSF. Furin itself is also a zymogen and has to enter the Golgi to become activated, which can be prevented by monensin, an inhibitor of transport to the Golgi (Vey et al., 1994). Thus, to investigate the activation of furin, cells were also pre-treated with monensin, prior to incubation with SMase D. **Figure 2** shows that both PMSF and monensin were effective in reducing the shedding of membrane markers by ADAMs, suggesting the involvement of serine proteinases and Furin in the mechanism of activation of ADAMs, on the keratinocytes membrane, after SMases D action. Reduction in shedding was not as effective as with the metalloprotease inhibitors.

**Figure 3:**
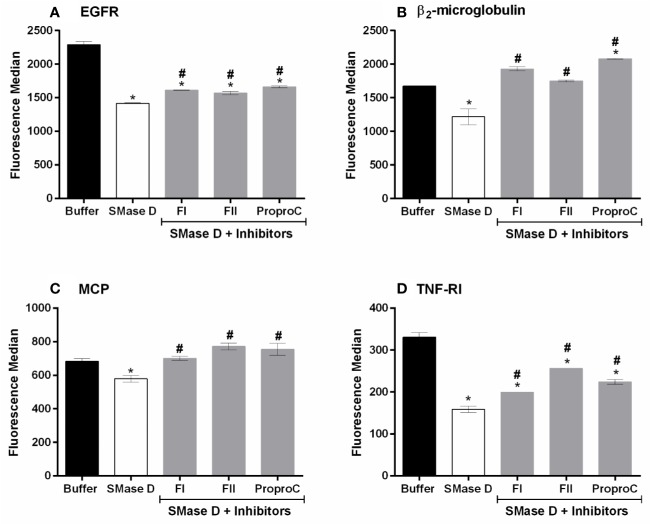
Proprotein convertases inhibitors partially prevent SMase D-induced shedding of surface markers. HaCaT cells were pre-incubated for 5 min with 20 µM of each inhibitor followed by SMase D (25 µg/ml) or buffer for 2 h. Expression of EGFR **(A)**, β2-microglobulin **(B)**, MCP **(C)**, and TNF-RI **(D)** was analyzed by flow cytometry. Data are presented as mean ± standard error of duplicates representative of two independent experiments. Statistically analyzed by one-way ANOVA followed by Tukey HSD test using GraphPad Prism 6.1 Software. (*) Significant difference compared to buffer (p < 0.05). (#) Significant difference in relation to SMase D (p < 0.05).

In an attempt to identify the proprotein convertases that participates in this process, the cells were treated with three different inhibitors of proprotein convertases groups, *i. e*., FI: specific furin inhibitor (Vey et al., 1995); FII: Furin, PACE4, and PC1 inhibitor (Cameron et al., 2000); and ProproC: Furin, PACE4, PC1/3, PC4, and PC5/6 inhibitor (Becker et al., 2012). **Figure 3** shows that the three inhibitors prevented the cleavage of membrane markers, to some extent, being most effective at inhibiting the release of cell surface β2-microglobulin and MCP. These results indicate that proproteins as furin, PACE4, PC1, PC7, and PC5B may be involved in the activation of ADAMs, induced by the action of SMase D in the membrane. The inhibitors did not have any effect on the cell surface expression of the molecules analyzed on the keratinocytes treated with buffer only, indicating that the observed effect was not due to an effect on the natural turnover of cell surface molecules (data not shown). The inhibitors or their solvents (DMSO, ethanol or PBS) did affect neither the cell viability nor the binding of the toxin to the cell membrane. Furthermore, the solvents of the inhibitors did not interfere with the cleavage of the cell surface markers induced by SMase D action on the cell membrane (data not shown).

### SMase D Modulates the Expression of ADAMs and Induces the Activation of ERK1/2 Signaling Pathway

**Figure 4:**
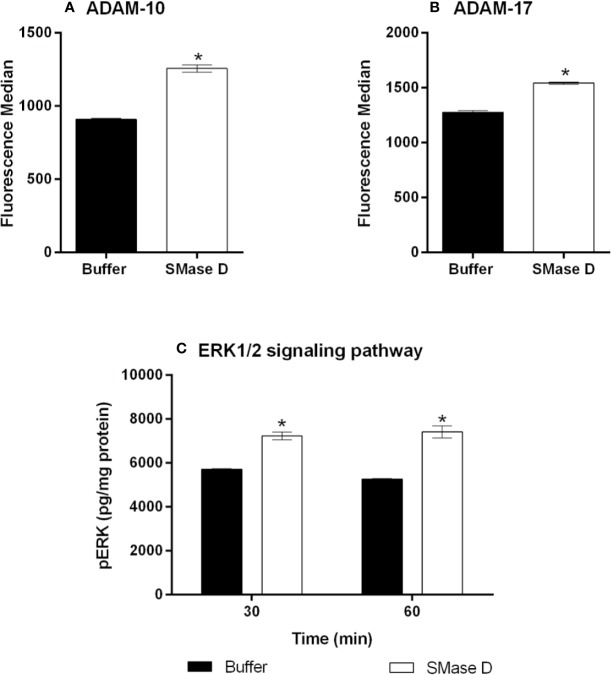
Expression of ADAM-10 and 17 and detection of ERK1/2 phosphorylation in human keratinocytes after treatment with SMase D. HaCaT cells were treated for 2 h with SMase D (25 µg/ml) or buffer and analyzed for the expression of ADAM-10 **(A)** and ADAM-17 **(B)** by flow cytometry. Alternatively, cells were treated with SMase D or buffer for 30 and 60 min and ERK1/2 phosphorylation was evaluated by using the EIA kit Titerzime Phospho-ERK1/2 **(C)**. Data are expressed as mean ± standard error of duplicates, representative of two independent experiments. Statistically analyzed by Two Way ANOVA followed by Tukey HSD test for the evaluation of ERK1/2 and analyzed by Student's t test for ADAMs expression, using the GraphPad Prism 5.1. (*) Significant difference compared to buffer (p < 0.05).

The results of the experiments, with ADAM-10 and ADAM-17 inhibitors, suggest an increase expression/function of these ADAMs and indeed, using flow-cytometry, we show that SMase D increases the detection/expression of ADAMs 10 and 17 on the cell surface (**Figures 4A, B**).

The shedding of surface molecules by ADAMs proteins may occur or increase due to specific signaling pathways such as MAPK and ERK (Xu et al., 2012). Based on this, we investigated the possible activation of ERK1/2 pathway after SMase D action on keratinocytes. **Figure 4C** shows that SMase D activated this signaling pathway, as demonstrated by an increased detection of ERK1/2 phosphorylation in SMase D-treated keratinocytes.

### SMase D Changes the Expression of Structural Lipid Rafts Components GM_1_ Ganglioside, Flotilin-1, and Caveolin-1 on Keratinocytes Membrane

**Figure 5:**
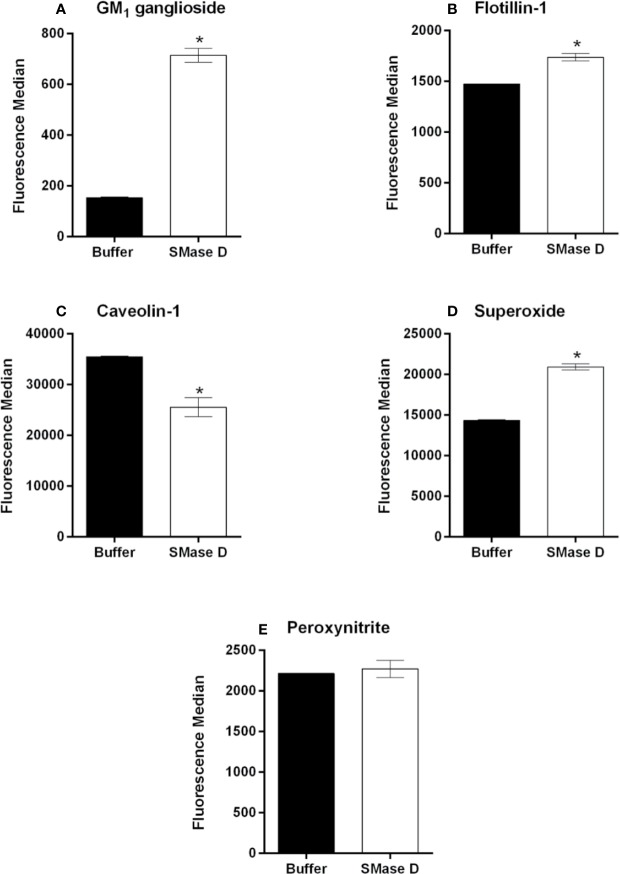
Analysis of GM_1_ ganglioside, flotillin-1, and caveolin-1 and ROS production in human keratinocytes, treated with SMase D. HaCaT cells were treated for 2 h with SMase D (25 µg/ml) or buffer and analyzed for the expression of GM_1_ ganglioside **(A)**, flotillin-1 **(B)**, and caveolin-1 **(C)** by flow cytometry or for the presence of superoxide (O_2_^• -^) **(D)** and peroxynitrite (ONOO^-^) **(E)**, by flow cytometry. Data are expressed as mean ± standard error of duplicates, representative of two independent experiments. Statistically analyzed by Student t test, using the GraphPad Prism 5.1. (*) Significant difference compared to buffer (p < 0.001).

Since the maturation of ADAM-17 occurs in lipid rafts and the mechanisms that regulate the hydrolytic activity of this and other proteases, involve proteins within the lipid rafts (Walev et al., 2000; Kojro et al., 2001; Matthews et al., 2003; von Tresckow et al., 2004; Zimina et al., 2007), we analyzed the effect of SMase D on the expression of the lipid raft markers GM_1_ ganglioside, flotillin-1, and caveolin-1 using flow cytometry. **Figures 5A, B** show that SMase D increased the detection or expression of GM_1_ ganglioside and flotillin-1, while it reduced the expression or detection of caveolin-1 (**Figure 5C**), an important component of a different lipid domain named as caveolae.

### SMase D Induces Superoxide Production in Human Keratinocytes

Considering that oxidative stress may contribute to the modification of lipid raft structural components (Park et al., 2009; Mougeolle et al., 2015) and that we observed a reduction in caveolin-1 expression after the SMase D treatment, we investigated the ability of SMase D to induce reactive oxygen and nitrogen species production. **Figure 5D** shows that SMase D induced a significant increase in superoxide production by human keratinocytes. However, the production of peroxynitrite was not affected by SMase D (**Figure 5E**).

### SMase D Binds to Lipid Rafts on Human Keratinocytes Membrane and Changes the Behavior of Other Proteins Present in the Microenvironment

**Figure 6:**
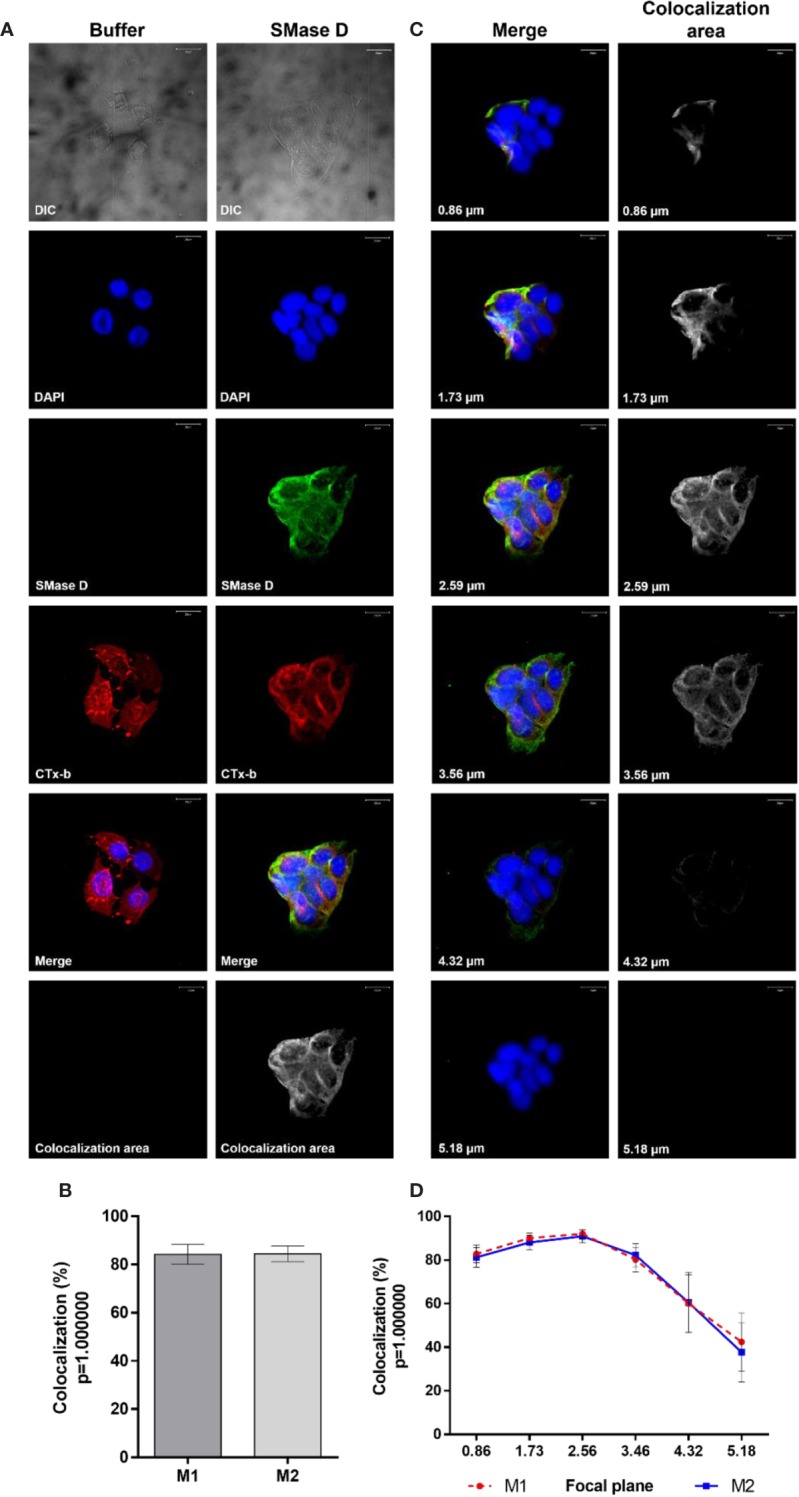
Colocalization of SMase D and Cholera Toxin subunit b in human keratinocytes membrane. HaCaT cells cultured on slides were treated, for 2 h, with buffer or SMase D (5 µg/ml). Binding of SMase D was analyzed using rabbit IgG anti-SMase D (1:200), followed by anti-rabbit secondary antibody conjugated with Alexa-488 (1:200). Lipid rafts were visualized using Cholera toxin subunit b-AlexaFluor 555 and the nuclei counterstained with DAPI and slides were analyzed by CLSM. Scale bars represent 20 µm. **(A)** Cells treated with buffer or SMase D, at focal plane of 2.59 µm. Colocalization areas are shown as grayscale images. **(B)** average percentage SMase D/CTx-b colocalization represent means ± SEM from, at least, 10 images in two independent experiments and three different focal plans. **(C)** Cells treated with SMase D at focal planes from 0.86 to 5.18 µm. Colocalization areas are shown as grayscale images. **(D)** Percentage of SMase D/CTx-b colocalization at the different focal planes analyzed. Data on the graphs represent means ± SEM from, at least, 10 images in two independent experiments and three different focal plans.

As structural components of lipid rafts were altered by the action of SMase D, we analyzed if the binding of SMase D on the cell membrane would colocalize with GM_1_ ganglioside, a marker of lipid rafts. Confocal microscopy, using SMase D specific antibodies and fluorescently labeled Choleratoxin b (CTx-b), which binds to GM_1_ was performed. **Figures 6A, B** showed that SMase D strongly colocalizes (about 85%) with GM_1_ ganglioside, with a Manders' colocalization coefficient of M1 = 0.84 and M2 = 0.85. Z-stack analysis showed that the bindings of SMase D and CTx-b occur mainly at the cell surface, since, as the depth of the focal planes in the Z-axis increased, the level of colocalization reduced (**Figures 6C, D**).

**Figure 7:**
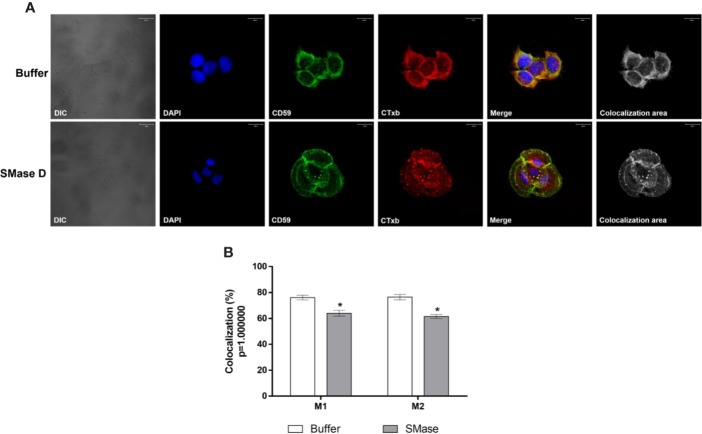
Colocalization of CD59 and Cholera Toxin subunit b in human keratinocytes, treated with SMase D. HaCaT cells were cultured on slides and treated for 2 h with buffer or SMase D (5µg/ml). Cells were stained with Moab anti-CD59 (1:250), followed by RAM-FITC (1:50). GM_1_ containing lipid rafts were visualized using Ctx-b/Alexa Fluor 555 and nuclei counterstained with DAPI and slides were analyzed by CLSM. Scale bars represent 20 µm. **(A)** Colocalization of CD59 and GM_1_ at the focal plane of 3.02 µm analyzed in cells treated with buffer or SMase D. Colocalization areas are shown as grayscale images. The graph **(B)** shows a comparison between the colocalization of CD59 and CTx-b, in cells treated with SMase D or buffer and represents the mean ± SEM from, at least, 10 images in two independent experiments and three different focal plans. Statistically analyzed by Two Way ANOVA followed by Tukey HSD test, using the GraphPad Prism 5.1. (*) Significant difference compared to buffer (p < 0.05).

**Figure 8:**
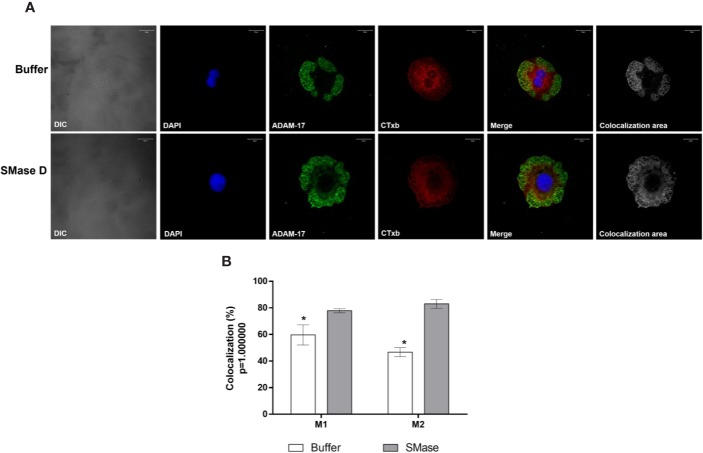
Colocalization of ADAM-17 and Cholera Toxin subunit b in human keratinocytes, treated with SMase D. HaCaT cells were cultured on slides and treated for 2 h with buffer or SMase D (5µg/ml). Cells were stained with Moab anti-ADAM-17 (20 µg/ml), followed by RAM-FITC (1:50). GM_1_ containing lipid rafts were visualized, using the CTx-b/Alexa Fluor 555 and the nuclei counterstained with DAPI and slides were analyzed by CLSM. Scale bars represent 20 µm. **(A)** Colocalization of ADAM-17 and GM_1_ at the focal plane of 3.02 µm analyzed in cells treated with buffer or SMase D. Colocalization areas are shown as grayscale images. **(B)** Comparison between the colocalization of ADAM-17 and Cholera Toxin subunit b in cells treated with SMase D or buffer and represent means ± SEM from at least 10 images in two independent experiments and three different focal plans. Statistically analyzed by Two Way ANOVA followed by Tukey HSD test, using the GraphPad Prism 5.1. (*) Significant difference compared to SMase D (p < 0.05).

To analyze the behavior of proteins known for their location or concentration of their activities in lipid rafts, we evaluated the colocalization of SMase D, CD59 (typically lipid raft associated) and ADAM-17, with GM_1_, in cells before and after SMase D incubation. **Figures 7A, B** show that SMase D significantly reduced the colocalization of CD59 and GM_1_ (Manders' colocalization coefficient M1 = 0.64; M2 = 0.61) when compared to cells treated with buffer alone (Manders' colocalization coefficient M1 = 0.76; M2 = 0.76). In contrast, **Figure 8** shows that SMase D significantly increased the colocalization of ADAM-17 and GM_1_ (Manders' colocalization coefficient M1 = 0.78; M2 = 0.83) when compared to cells treated with buffer (Manders' colocalization coefficient M1 = 0.60; M2 = 0.47).

## Discussion

Various interventions have been proposed as a treatment for loxoscelism, however, a definitive and fully effective therapy has not yet been established. A better understanding of the molecular mechanism of venom/toxins action is important to the establishment of more effective therapeutic approaches for the *Loxosceles* spider envenomation.

In previous studies, using a broad-spectrum metalloproteinase inhibitor Galardin (GM6001), we demonstrated that *Loxosceles* venom/SMases D activated metalloproteinases of the Adamalysin family on nucleated cells surface resulting in cleavage of various transmembrane anchored molecules (van den Berg et al., 2002; van den Berg et al., 2012). Here, we show that in addition to Galardin, specific ADAM-10 and -17 inhibitors significantly reduced the cleavage of the cell surface markers EGFR, β2-microglobulin and MCP. As also previously described by us (van den Berg et al., 2002), CD59 was not affected by the action of SMase D (**Figure 1B**). Furthermore, we show here for the first time that TNF-RI is also cleaved by the indirect action of SMase D on keratinocytes. Thus, data obtained with these inhibitors indicate that both ADAM-10 and -17 are activated by SMase D and contribute to the cleavage/shedding of cell surface molecules. Combined inhibition of these enzymes did not provide a complete inhibition of the shedding, suggesting that other ADAMs may be involved. According to Ari-Pekka et al. (Huovila et al., 2005), while ADAM-10 and -17 are the main sheddases, several other ADAMs contribute to the shedding of membrane bound proteins.

The results presented here show that proteins cleaved after the action of the SMases D are part of the group of specific substrates of ADAM-10 and -17. Shedding of surface molecules by ADAMs proteins may occur or increase in response to cellular stimulation with phorbol esters (Müllberg et al., 2000), bacterial toxins (Walev et al., 1996), apoptotic stimuli (Chalaris et al., 2007), and activation of MAPK and ERK (Xu et al., 2012). Our results showing SMase D-induced activation of MAPK ERK1/2 signaling pathway (**Figure 4C**) in keratinocytes, may suggest that the activation of this pathway may contribute to the ADAMs activity.

The metalloprotease domain of ADAMs is protected by a pro-domain in the inactive zymogens, which is removed by proprotein convertases (PCs) such as furin, PC7, PC5/6B, SKI-1. These PCs, which are serine proteases (Seidah, 2006; Klein and Bischoff, 2011), are activated themselves during transport through the Golgi. Inhibiting serine protease activity with the broad-spectrum inhibitor PMSF and inhibiting Golgi transport, using monensin, resulted in a partial (EGFR, TNF-RI) to complete (MCP and β_2_-microglobulin) inhibition of SMase D-induced shedding, suggesting the involvement of proprotein convertases (**Figure 2**). Using more specific inhibitors of the proprotein convertases, including furin, their roles were confirmed, especially in the shedding of MCP and β_2_-microglobulin, which cleavages were completely inhibited (**Figure 3**). We thus show here for the first time the participation of proprotein convertases in the SMase D-induced shedding of cell surface molecules.

The furin specific inhibitor FI showed, in most cases, to be equally efficient to the broader spectrum inhibitors FII and ProproC, suggesting that furin is the main proprotein convertase activated and involved in this process. However, in the case of EGFR and TNF-RI, we did not observe a complete inhibition and other mechanisms may contribute to the shedding of these molecules.

Lipid raft disruption has been shown to increase shedding by ADAMs-10 and -17 (Matthews et al., 2003; von Tresckow et al., 2004). Tellier et al. (2006) showed that the zymogen pro-domain of ADAM-17 is cleaved by furin in lipid rafts, which results in concentration of the shedding activity of ADAM-17 in lipid rafts and inhibition of ADAM-17 resulted in increase in its substrates within the rafts. Our results presented here also showed that SMase D-induced cleavage of several cell surface proteins, in the cell membrane, was prevented by ADAMs inhibitors. Corroborating these findings, our confocal microscopy data showed that SMase D changed the behavior of molecules located in the lipid rafts, resulting in an increased lipid raft colocalization index of ADAM-17 and a decreased CD59 colocalization (**Figures 8** and **7**, respectively).

Diaz and colleagues (2005) showed that sphingomyelinase C from *Staphylococcus aureus*, altered the properties of lipid rafts in peripheral blood derived mononuclear cells, resulting in a concomitant reduction of sphingomyelin and cholesterol content of the rafts. We have tried to emulate the action of SMase D and the subsequent cleavage of various cell surface molecules with commercial purified Sphingomyelinase C and with ceramide-1-phosphate, but while the Sphingomyelinase C did not induce cleavage of cell surface molecules, the ceramide-1-phosphate was toxic to the cells and results were inconclusive (unpublished observations). Therefore our observation that SMase D changes the lipid raft composition and stability and increases the activity of ADAMs, suggest that this is a unique property of this SMase D and also reveals the importance of these microdomains in controlling this process, as demonstrated by Tellier et al. (2006).

Since the shedding process by ADAMs occurs in the lipid rafts following “perturbation” of the cell membrane environment, we evaluated the expression of the major component of lipid rafts GM_1_ ganglioside, after treatment with SMase D. We observed that the ganglioside detection increased significantly after the action of SMase D, suggesting possible lipid raft disruption leading to an enhanced binding of CTx-b to GM_1_ ganglioside, probably due to increased molecule accessibility (Slaughter et al., 2003).

There are two types of rafts: those containing the structural protein caveolin-1 that form caveolae, and those that lack this protein but express two different raft-specific proteins, called flotillin-1 and 2 (Volonté et al., 1999). As both caveolar and non-caveolar rafts are highly enriched in sphingolipids and glycosphingolipids, they are also known as glycolipid-enriched microdomains. These rafts are also highly enriched in gangliosides, especially GM_1_ which has almost been exclusively identified in these structures (Cremesti et al., 2002). Thus, we have demonstrated that SMase D is capable of interfering with both types of rafts, since a reduction in caveolin-1 expression/detection and increase in flotilin-1 was observed in cells treated with SMase D. Mougeolle et al. (2015) demonstrated that oxidative stress induces caveolin-1 degradation. We have previously shown that SMase D induces oxidative stress in leukocytes with production of superoxide and peroxynitrite (Manzoni-de-Almeida et al., 2018), and corroborating this data, we show here that SMase D induces superoxide production in human keratinocytes (**Figures 5C, D**). Regarding the flotillin-1, its increase may indicate an augmented synthesis and recruitment of raft components to the membrane after degradation or perturbation. In the absence of caveolins, flotillins has been shown to assume the role of a structural protein assisting lipid rafts assembly (Slaughter et al., 2003).

To confirm the action of SMase D on lipid rafts, we sought to analyze the possible toxin binding to the microdomains, which were indirectly visualized by Cholera toxin labeling, which binds to GM_1_ gangliosides. We observed a high colocalization index between SMase D in the membrane and the GM_1_ ganglioside, suggesting that the SMase D acts on the membrane and preferably in these microdomains.

In conclusion, we have elucidated more of the mechanism by which SMase D exerts its actions and our observation that SMase D changes the rafts dynamics leading to activation of proproteases such as furin and, consequently, the metalloproteases ADAM-10 and -17, opens up pathways for novel therapeutic interventions to prevent and treat systemic and local pathologies after *Loxosceles* spider envenomation.

## Data Availability Statement

All datasets generated for this study are included in the article/supplementary material.

## Author Contributions

PL conceived the project, performed all the experiments, analyzed the data, discussed the results, and wrote the manuscript. CB discussed the results and wrote the manuscript. DT conceived the project, discussed the results, and wrote the manuscript. All authors read, revised, and approved the submitted manuscript.

## Funding

This work was supported by São Paulo Research Foundation (FAPESP) to Centre of Toxins, Immune Response and Cell Signalling (CeTICS) (grant 2013/07467-1) and to PL (grant 2015/17053-5) and CNPq (grant 162570/2013-9). DT is recipient of CNPq Research Productivity Fellowship. The funding agencies had no influence on study design, data interpretation, or form of the manuscript.

## Conflict of Interest

The authors declare that the research was conducted in the absence of any commercial or financial relationships that could be construed as a potential conflict of interest.
